# Variation in the *SLC23A1* gene does not influence cardiometabolic outcomes to the extent expected given its association with l-ascorbic acid^1^,^2^,^3^,^4^

**DOI:** 10.3945/ajcn.114.092981

**Published:** 2014-11-19

**Authors:** Kaitlin H Wade, Nita G Forouhi, Derek G Cook, Paul Johnson, Alex McConnachie, Richard W Morris, Santiago Rodriguez, Zheng Ye, Shah Ebrahim, Sandosh Padmanabhan, Graham Watt, K Richard Bruckdorfer, Nick J Wareham, Peter H Whincup, Stephen Chanock, Naveed Sattar, Debbie A Lawlor, George Davey Smith, Nicholas J Timpson

**Affiliations:** 1 From the Medical Research Council (MRC) Integrative Epidemiology Unit (KHW, DAL, GDS, and NJT) and the School of Social and Community Medicine (KHW, NGF, SR, DAL, GDS, and NJT), University of Bristol, Bristol, United Kingdom; the MRC Epidemiology Unit, University of Cambridge School of Clinical Medicine, Institute of Metabolic Science, Cambridge, United Kingdom (NGF, ZY, and NJW); the Division of Community and Health Sciences, St. George's University of London, London, United Kingdom (DGC and PHW); the Robertson Centre for Biostatistics, Glasgow, United Kingdom (PJ and AM); the Department of Primary Care & Population Health (RWM), the Department of Structural and Molecular Biology, University College London (KRB), London, United Kingdom (RWM); the London School of Hygiene and Tropical Medicine, London, United Kingdom (SE); the British Heart Foundation Glasgow Cardiovascular Research Centre, Faculty of Medicine (SP and NS) and General Practice and Primary Care, Division of Community Based Sciences (GW), University of Glasgow, Glasgow, United Kingdom; and the Division of Cancer Epidemiology and Genetics, National Cancer Institute, Bethesda, MD (SC).

**Keywords:** l-ascorbic acid, cardiometabolic traits, confounding, genetic variants, reverse causation

## Abstract

**Background:** Observational studies showed that circulating l-ascorbic acid (vitamin C) is inversely associated with cardiometabolic traits. However, these studies were susceptible to confounding and reverse causation.

**Objectives:** We assessed the relation between l-ascorbic acid and 10 cardiometabolic traits by using a single nucleotide polymorphism in the solute carrier family 23 member 1 (*SLC23A1*) gene (rs33972313) associated with circulating l-ascorbic acid concentrations. The observed association between rs33972313 and cardiometabolic outcomes was compared with that expected given the rs33972313-l-ascorbic acid and l-ascorbic acid–outcome associations.

**Design:** A meta-analysis was performed in the following 5 independent studies: the British Women's Heart and Health Study (*n* = 1833), the MIDSPAN study (*n* = 1138), the Ten Towns study (*n* = 1324), the British Regional Heart Study (*n* = 2521), and the European Prospective Investigation into Cancer (*n* = 3737).

**Results:** With the use of a meta-analysis of observational estimates, inverse associations were shown between l-ascorbic acid and systolic blood pressure, triglycerides, and the waist-hip ratio [the strongest of which was the waist-hip ratio (−0.13-SD change; 95% CI: −0.20-, −0.07-SD change; *P* = 0.0001) per SD increase in l-ascorbic acid], and a positive association was shown with high-density lipoprotein (HDL) cholesterol. The variation at rs33972313 was associated with a 0.18-SD (95% CI: 0.10-, 0.25-SD; *P* = 3.34 × 10^−6^) increase in l-ascorbic acid per effect allele. There was no evidence of a relation between the variation at rs33972313 and any cardiometabolic outcome. Although observed estimates were not statistically different from expected associations between rs33972313 and cardiometabolic outcomes, estimates for low-density lipoprotein cholesterol, HDL cholesterol, triglycerides, glucose, and body mass index were in the opposite direction to those expected.

**Conclusions:** The nature of the genetic association exploited in this study led to limited statistical application, but despite this, when all cardiometabolic traits were assessed, there was no evidence of any trend supporting a protective role of l-ascorbic acid. In the context of existing work, these results add to the suggestion that observational relations between l-ascorbic acid and cardiometabolic health may be attributable to confounding and reverse causation.

## INTRODUCTION

An understanding of the associations between l-ascorbic acid (vitamin C) and cardiometabolic traits is of particular importance to public health because of the potential to modify this target. However, evidence as to the likely benefit of such an intervention has been mixed. Several prospective cohort studies suggested that higher concentrations of circulatory l-ascorbic acid are associated with a favorable cardiometabolic risk profile. This profile includes reduced blood pressure, glucose, cholesterol, and insulin (1–3) and reduced risk of cardiovascular diseases (CVDs)^5^ and all-cause mortality (4–9).

However, these observational studies may have been biased because of confounding or suffer reverse causation, whereby an undiagnosed disease may have influenced dietary intake or circulating concentrations of l-ascorbic acid (10). Therefore, randomized controlled trials (RCTs) were undertaken and provided some evidence of beneficial effects of l-ascorbic acid supplementation or high dietary intake of fruit and vegetables (naturally high in l-ascorbic acid) on cardiometabolic traits including LDL cholesterol and HDL cholesterol, triglycerides, insulin, glucose, and blood pressure (11, 12) and risk of coronary artery disease and hypertension during follow-up (13–16).

Despite this evidence, other estimates of the causal association between l-ascorbic acid and cardiometabolic health were not consistent (17–22). A long-term RCT of vitamin supplementation on the prevention of CVD in >14,500 men showed that, after 8 y follow up, there was no evidence that l-ascorbic acid reduced risk of major cardiometabolic events (HR: 0.99; 95% CI: 0.89, 1.11; *P* = 0.91) or CVD mortality (HR: 1.02; 95% CI: 0.85, 1.21; *P* = 0.86) (18). Similarly, in an RCT of vitamin supplementation in >8000 women at high risk of CVD, no overall effects were seen between l-ascorbic acid on later myocardial infarction, stroke, coronary revascularization, or CVD death (risk ratio: 1.02; 95% CI: 0.92, 1.13; *P* = 0.71) (19).

The discovery of the association between the variation at rs33972313 and circulating l-ascorbic acid (23) provides an opportunity to use this genetic variant as an instrumental variable (IV) to test putatively causal relations between l-ascorbic acid and cardiometabolic outcomes (24–27). The aim of this study was to explore the utility of the variation at the rs33972313 single nucleotide polymorphism (SNP) to reexamine potentially causal effects of l-ascorbic acid on a panel of cardiometabolic traits in a large collection of European samples.

## SUBJECTS AND METHODS

Data from the following 5 independent studies including 18,802 individuals were available: the British Women's Heart and Health Study, the MIDSPAN study, the Ten Towns study, the British Regional Heart Study (BRHS), and the European Prospective Investigation into Cancer (EPIC) study. Related individuals in the MIDSPAN study were excluded to remove nonindependent observations. Full details of individual studies, their ethical approvals, DNA extraction, genotyping, and measurements of l-ascorbic acid concentrations and cardiometabolic variables were previously published (23) and are provided in the **Supplemental Material**. Across the 5 cohorts, 15,959 individuals had data on both genotype and l-ascorbic acid. In total, full genotypic data and information on l-ascorbic acid and cardiometabolic outcomes were available for 10,553 participants from the British Women's Heart and Health Study (*n* = 1833), the MIDSPAN study (*n* = 1138), the Ten Towns study (*n* = 1324), the BRHS (*n* = 2521), and the EPIC study (*n* = 3737).

### Cardiometabolic outcomes

Eight continuous quantitative traits were shared across the 5 independent studies. Information was available for systolic blood pressure (SBP; *n* = 18,449), diastolic blood pressure (DBP; *n* = 18,449), cholesterol (*n* = 18,082), HDL cholesterol (*n* = 17,486), triglycerides (*n* = 16,956), glucose (*n* = 16,550), BMI (*n* = 18,438), and the waist-hip ratio (WHR; *n* = 18,410). The remaining 2 variables were LDL cholesterol and hypertension. LDL cholesterol was derived by using Friedewald's equation for individuals with triglyceride concentrations <4 mmol/L (28) within all 5 cohorts (*n* = 17,350). Hypertension was defined as SBP ≥ 140 mmHg and DBP ≥ 90 mmHg and used as a binary outcome.

### Genetic variation

We previously showed that the rs33972313 SNP at the solute carrier family 23 member 1 (*SLC23A1*) locus, which encodes the sodium-dependent l-ascorbic acid transporter-1, was reliably associated with circulating l-ascorbic acid concentrations (23). The rs33972313 variant exists in European populations with a minor allele frequency (MAF) ∼0.06 (merged HapMap phases I–III, release 28). Each additional minor allele of rs33972313 was associated with a 5.98-μmol/L (95% CI: 3.73-, 8.23-μmol/L; *P* = 2.0 × 10^−7^) lower circulating l-ascorbic acid concentration, and as such, this SNP was selected as a genetic instrument for circulating l-ascorbic acid in this study. Genotyping and appropriate quality control was undertaken with methods specific to each study (Supplemental Material). Full genetic information was available for 16,841 individuals from the 5 studies.

### Statistical analysis

All analyses were performed with STATA version 12 software (StataCorp LP). An inverse rank transformation was used for l-ascorbic acid and continuous cardiometabolic traits to harmonize data across contributing studies before applied analyses and take into account systematic differences in the absolute value of traits because of differing study protocols [as seen previously (23)]. *z* scores of transformed continuous traits were generated to provide per SD effect estimates for the purposes of interpretation. The association between each quantitative trait and l-ascorbic acid was examined by using linear regression of the trait *z* score against l-ascorbic acid *z* score. The association between rs33972313 and each quantitative trait (including l-ascorbic acid) was examined by using a linear regression of the trait *z* score against genotype. Logistic regression was used to investigate associations between both the rs33972313 genotype and l-ascorbic acid and hypertension risk expressed as ORs.

As each additional minor allele of rs33972313 was associated with a decrease in l-ascorbic acid, genotypes at this SNP were coded in an additive model as 0 (minor homozygotes), 1 (heterozygotes), and 2 (major homozygotes) to give an l-ascorbic acid increase per effect allele. Hardy-Weinberg equilibrium was tested for rs33972313 with the Stata command genhw.

### Meta-analysis

Summary statistics were pooled by using a meta-analysis with a random-effects model to account for differences between cohorts and methods (by using the *random* option for the Stata command metan). The *I*^2^ statistic was used to estimate heterogeneity between studies (29). A sensitivity analysis was performed by excluding subjects known to be taking an antihypertensive medication.

### Calculating expected effect estimates for associations between *SLC23A1* genotype and cardiometabolic outcomes

**FIGURE 1:**
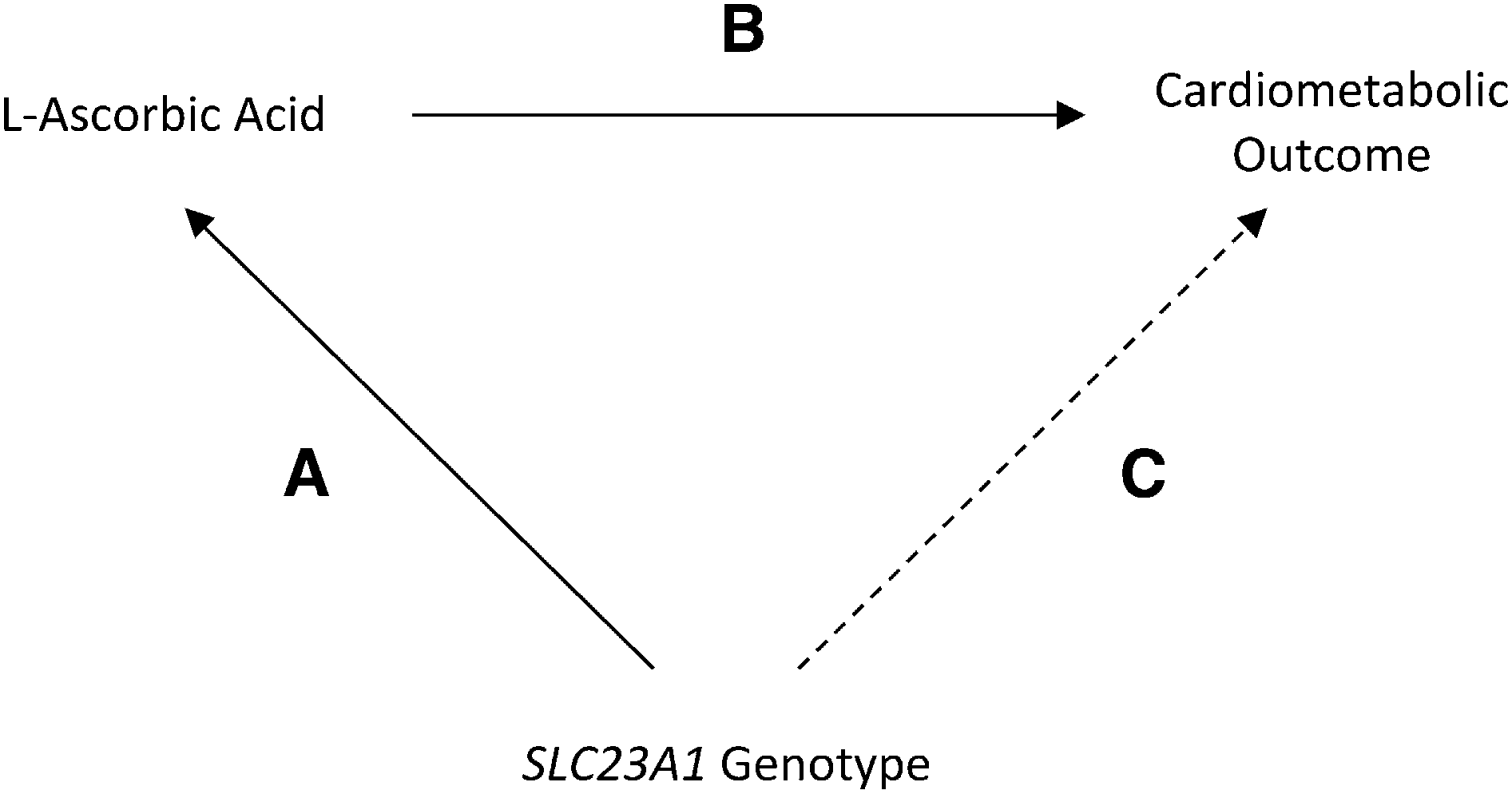
Triangulation approach used to estimate the effect size of the *SLC23A1*-cardiometabolic outcome association (C) given the association between *SLC23A1* and l-ascorbic acid (A) and observed associations between l-ascorbic acid and the outcomes (B). We hypothesized that associations observed between the *SLC23A1* genotype and cardiometabolic outcomes (dotted line) would be mediated by l-ascorbic acid (i.e., C = A × B; solid lines). Therefore, effect estimates should be expected to reflect both the *SLC23A1*–l-ascorbic acid association and l-ascorbic acid–cardiometabolic outcome associations. *SLC23A1*, solute carrier family 23 member 1.

Despite a relatively strong relation between the variation at rs33972313 and l-ascorbic acid (which, in isolation, remains the best instrument for l-ascorbic acid at this locus), the MAF at this variant was such to preclude the use of a formal IV approach because of weak instrumentation (as assessed by using an *F* test in the first of a 2-stage least-squares analysis) (30, 31). As a result, a triangulation approach (32) was used to estimate expected effect estimates (95% CIs) for associations between the rs33972313 genotype and cardiometabolic outcomes (**Figure 1**). Under the assumption that there is a true causal association between l-ascorbic acid and each cardiometabolic outcome, and because rs33972313 had an independent association with l-ascorbic acid, it was predicted that the *SLC23A1*-outcome association (Figure 1C) would be proportional to the relation of both the *SLC23A1*–l-ascorbic acid association (Figure 1A) and each l-ascorbic acid–outcome association (Figure 1B) (Supplemental Material). The method assumes that the portion of variance explained in l-ascorbic acid by rs33972313 is independent of other factors, and therefore, the genetic variant provides an unconfounded instrument for l-ascorbic acid that is not susceptible to reverse causation.

To generate the expected SD change in each cardiometabolic trait per SD increase in l-ascorbic acid, the effect estimate of each l-ascorbic acid–trait association was multiplied by the magnitude of the effect of rs33972313 on l-ascorbic acid. SEs for expected estimates were calculated by using the Taylor series expansion of the product of observed and expected estimates (33). Expected ORs and SEs for hypertension risk were calculated by using the same method on the log(OR) scale and back transformed onto the OR scale by exponentiating calculated expected estimates. Observed and expected estimates for each *SLC23A1*-outcome association were compared by using the methodology previously reported (32) (Supplemental Material). If the test of the comparison of observed and expected associations gave evidence of a statistical difference between effect estimates (*P* < 0.05), this result provided no evidence of a causal association between l-ascorbic acid and the cardiovascular outcome. To assess the causal association, cardiometabolic outcomes were analyzed individually and in combination.

## RESULTS

Mean (±SD) l-ascorbic acid concentrations ranged between 30.21 ± 27.55 μmol/L in the BRHS to 52.70 ± 20.40 μmol/L in the EPIC study (**Table 1**). Summary statistics of all cardiometabolic traits and the prevalence of hypertension are presented in Table 1. Across all studies, rs33972313 was observed at an average MAF of 0.04 with no overall departure from the Hardy-Weinberg equilibrium within all studies except the MIDSPAN study (*P* = 0.0002) (**Table 2**).

**TABLE 1:** Descriptive characteristics for each of the included cohorts^1^

	BWHHS (*n* = 4286)		MIDSPAN (*n* = 1477)		Ten Towns (*n* = 1531)		BRHS (*n* = 3945)		EPIC (*n* = 7563)	
Variables	*n*	Values	*n*	Values	*n*	Values	*n*	Values	*n*	Values
Women, %	4286	100	1477	54.91	1531	44.74	3945	0	7563	50.64
Age, y	4284	68.88 ± 5.51^2^	1477	45.39 ± 6.04	1531	15.06 ± 0.58	3945	68.74 ± 5.49	7563	59.50 ± 9.22
l-ascorbic acid, μmol/L	3606	43.25 ± 28.04	1364	49.20 ± 25.27	1531	50.36 ± 23.92	3811	30.21 ± 27.55	7106	52.70 ± 20.40
SBP, mm Hg	3964	147.13 ± 25.23	1464	127.53 ± 15.69	1527	120.82 ± 12.96	3942	142.78 ± 19.56	7552	136.08 ± 18.25
DBP, mm Hg	3964	79.39 ± 11.66	1464	74.75 ± 11.17	1527	66.91 ± 7.34	3942	81.42 ± 12.80	7552	83.00 ± 11.16
WHR	3946	0.82 ± 0.07	1462	0.84 ± 0.09	1528	0.76 ± 0.06	3918	0.95 ± 0.06	7556	0.86 ± 0.09
BMI, kg/m^2^	3957	27.62 ± 5.01	1467	26.19 ± 4.52	1525	20.93 ± 3.64	3926	26.88 ± 3.68	7563	26.48 ± 3.90
Cholesterol, mmol/L	3851	6.64 ± 1.21	1452	5.29 ± 0.97	1523	4.22 ± 0.72	3927	6.31 ± 1.03	7329	6.18 ± 1.18
Glucose, mmol/L	3829	6.06 ± 1.66	1454	5.35 ± 1.54	1500	5.09 ± 0.81	3925	5.56 ± 1.25	5842	4.51 ± 2.22
HDL cholesterol, mmol/L	3845	1.66 ± 0.45	1279	1.42 ± 0.37	1523	1.45 ± 0.30	3813	1.15 ± 0.25	7026	1.40 ± 0.42
LDL cholesterol,^3^ mmol/L	3762	4.14 ± 1.09	1276	3.20 ± 0.87	1523	2.32 ± 0.62	3832	3.90 ± 0.98	6957	3.65 ± 1.04
Triglycerides, mmol/L	3851	1.87 ± 1.19	1448	1.60 ± 1.26	1523	1.01 ± 0.41	2805	2.03 ± 1.29	7329	1.89 ± 1.15
Prevalent hypertension^4^	2226	30.14	1477	13.20	1404	0.28	3942	53.53	6062	28.04

1 BRHS, British Regional Heart Study; BWHHS, British Women's Heart and Health Study; DBP, diastolic blood pressure; EPIC, European Prospective Investigation into Cancer; SBP, systolic blood pressure; WHR, waist-hip ratio.

2 Mean ± SD (all such values).

3 Derived by using Friedewald's equation (with the exclusion of individuals with triglyceride concentrations ≥4 mmol/L) (28).

4 Hypertension was defined as SBP ≥ 140 mm Hg and DBP ≥ 90 mm Hg.

**TABLE 2:** SNP description showing genotypic frequencies, MAF, and HapMap figures of MAF for rs33972313^1^

		Genotype frequencies, %					
Study	*n*	Minor homozygote (AA)	Heterozygote (AG)	Major homozygote (GG)	MAF	Hardy-Weinberg equilibrium *P*	HapMap
BWHHS	3719	0.16	6.29	93.55	0.03	0.30	0.06
MIDSPAN	1379	0.73	7.32	91.95	0.04	0.0002	
Ten Towns	1477	0.14	6.77	93.09	0.04	0.70	
BRHS	3870	0.10	6.64	93.26	0.03	0.79	
EPIC	6396	0.16	6.93	92.92	0.04	0.59

1 BRHS, British Regional Heart Study; BWHHS, British Women's Heart and Health Study; EPIC, European Prospective Investigation into Cancer; MAF, minor allele frequency; SNP; single nucleotide polymorphism.

### Association between *SLC23A1* and l-ascorbic acid

A pooled analysis of the relation between rs33972313 and circulating l-ascorbic acid concentrations showed a 0.23-SD (95% CI: 0.12-, 0.35-SD; *P* = 8.26 × 10^−5^) increase in l-ascorbic acid per effect allele. Consistent with results previously reported (23), there was a high level of heterogeneity in the meta-analysis of all studies (*I^2^*: 71.9%; 95% CI: 29%, 89%; *P* = 0.007) (**Supplemental Figure S1**), which was reduced only by the exclusion of the EPIC study (*I^2^* = 0%; *P* = 0.99). Because of this result and to allow for additional applied analyses, the EPIC study was removed from main analyses and treated as an independent replication study (although random-effects models were still used, and sensitivity meta-analyses were undertaken that included the EPIC study).

The meta-analysis with the exclusion of the EPIC study showed a 0.18-SD (95% CI: 0.10-, 0.25-SD; *P* = 3.34 × 10^−6^) increase in l-ascorbic acid per effect allele (**Table 3**, **Supplemental Figure S2**). Within the EPIC study, each additional effect allele was associated with a 0.40-SD (95% CI: 0.31-, 0.50-SD; *P* = 2.73 × 10^−17^) increase in l-ascorbic acid (Table 3).

**TABLE 3:** Associations between rs33972313 and l-ascorbic acid in the 4 included studies (*n* = 9946)^1^

		l-ascorbic acid per allele variation, μmol/L					
Study	*n*	Minor homozygote (AA)	Heterozygote (AG)	Major homozygote (GG)	Per allele effect on standardized l-ascorbic acid	*P*	Variance explained by SNP, %
BWHHS	3438	52.61 (17.94, 87.28)^2^	38.61 (35.07, 42.14)	43.69 (42.72, 44.66)	0.17 (0.04, 0.30)	0.01	0.19
MIDSPAN	1291	39.30 (24.08, 54.52)	45.02 (40.63, 49.42)	49.68 (48.22, 51.14)	0.16 (−0.01, 0.34)	0.07	0.40
Ten Towns	1477	64.0 (−520.49, 648.49)	44.86 (40.99, 48.73)	50.64 (49.36, 51.92)	0.20 (0.01, 0.40)	0.04	0.28
BRHS	3740	21.53 (6.43, 36.62)	27.55 (24.25, 30.85)	30.31 (29.41, 31.21)	0.18 (0.05, 0.30)	0.01	0.21
Meta-analysis	9946	33.35 (19.70, 46.99)	38.94 (30.55, 47.34)	43.57 (33.77, 53.37)	0.18 (0.10, 0.25)	3.34 × 10^−6^	0.28
EPIC	6013	36.50 (27.24, 45.76)	46.63 (45.06, 48.20)	54.74 (54.21, 55.28)	0.40 (0.31, 0.50)	2.73 × 10^−17^	1.18

1 BRHS, British Regional Heart Study; BWHHS, British Women's Heart and Health Study; EPIC, European Prospective Investigation into Cancer; SNP, single nucleotide polymorphism.

2 Mean; 95% CI in parentheses (all such values).

### Associations between l-ascorbic acid and cardiometabolic outcomes and confounders

Meta-analyses of observational estimates suggested that circulating concentrations of l-ascorbic acid were inversely associated with SBP, triglycerides, and the WHR (**Table 4**, **Supplemental Figures S3** and **S4**), the strongest of which was the WHR [−0.13-SD change (95% CI: −0.20-, −0.07-SD change; *P* = 0.0001) per SD increase in l-ascorbic acid]. There was also evidence of a positive association between l-ascorbic acid and HDL cholesterol (Table 4, Supplemental Figures S3 and S4). Within the EPIC study, there were strong inverse associations between l-ascorbic acid and SBP, DBP, cholesterol, LDL cholesterol, triglycerides, glucose, BMI, and the WHR and a positive association between l-ascorbic acid and HDL cholesterol (**Supplemental Table S1**). The OR for hypertension risk was 0.75 (95% CI: 0.71, 0.80; *P* = 2.48 × 10^−21^) per SD increase in l-ascorbic acid. The inclusion of the EPIC study within meta-analyses gave similar results (**Supplemental Figure S5**). In meta-analyses of observational estimates, there was evidence of heterogeneity (all *I*^2^ > 60%) (Table 4, Supplemental Figure S4).

**TABLE 4:** Meta-analysis of associations of l-ascorbic acid with cardiometabolic outcomes within 4 cohorts^1^

Phenotype	*n*	Observed change in outcome per SD change in l-ascorbic acid^2^	*P*	*I*^2^, %
SBP	10,274	−0.06 (−0.10, −0.02)	0.002	70.0
DBP	10,274	−0.05 (−0.13, 0.02)	0.14	92.5
Cholesterol	10,247	−0.001 (−0.05, 0.04)	0.95	78.8
HDL cholesterol	9985	0.06 (0.0003, 0.12)	0.05	88.9
LDL cholesterol	9936	0.005 (−0.04, 0.05)	0.82	76.5
Triglycerides	9146	−0.09 (−0.16, −0.02)	0.02	91.7
Glucose	10,202	−0.04 (−0.09, 0.01)	0.12	82.9
BMI	10,224	−0.05 (−0.11, 0.01)	0.09	88.9
WHR	10,236	−0.13 (−0.20, −0.07)	0.0001	91.2
Hypertension^3^	8592	0.93 (0.83, 1.03)	0.17	63.2

1 *P* values are from a random-effects meta-analysis of linear regression coefficients estimated within each study for each phenotype *z* score (on the inverse rank scale) against l-ascorbic acid *z* score (inverse rank scale). *I*^2^ is the percentage of the total variance in study estimates that was attributable to between-study heterogeneity (29). DBP, diastolic blood pressure; SBP, systolic blood pressure; WHR, waist-hip ratio

2 All values are means; 95% CIs in parentheses. Values are for effect sizes per SD increase in l-ascorbic acid and meta-analysis *P* values. All continuous traits were inverse-rank transformed before calculation of the *z* score.

3 Estimates were calculated on the log(OR) scale and exponentiated to give an expected OR estimate for hypertension risk.

Across all studies, l-ascorbic acid was associated with smoking status, alcohol intake, physical activity, and socioeconomic position alongside obesity, insulin, C-reactive protein, IL-6, and urate (where available) (**Supplemental Table S2**). Adjustment for potentially confounding factors including age, sex (where appropriate), smoking and alcohol use, physical activity, and measures of socioeconomic position made little difference to observational associations (results available from authors upon request).

### Association between *SLC23A1* and cardiometabolic outcomes and confounders

Meta-analyses from 4 studies of the observed change in cardiometabolic outcomes per effect allele showed no substantive evidence for an association between rs33972313 and any cardiometabolic outcome (**Table 5**, **Supplemental Figures S6** and **S7**). There was little evidence of between-study heterogeneity for a majority of the cardiometabolic outcomes [*I^2^* ≤ 15% for all cardiometabolic outcomes except HDL (39.2%) and triglycerides (64%)]. Within the EPIC study, there were no associations between any of the cardiometabolic outcomes and rs33972313 (**Supplemental Table S3**), and the inclusion of the EPIC study within the meta-analysis gave results consistent with those of the main analysis (**Supplemental Figure S8**), but heterogeneity was introduced. Across all cohorts, rs33972313 was not associated with any of the potentially confounding factors (**Supplemental Table S4**).

**TABLE 5:** Meta-analysis of associations of cardiometabolic outcomes with rs33972313 and with l-ascorbic acid within 4 cohorts^1^

		Change in outcome *z* score per effect allele^2^				
Phenotype	*n*	Expected	Observed	*P*-rs33972313 association with outcome^3^	*I*^2^, %	*P*-difference between observed and expected
SBP	10,402	−0.01 (−0.02, −0.003)	−0.02 (−0.09, 0.06)	0.63	0	0.84
DBP	10,402	−0.01 (−0.02, 0.004)	−0.02 (−0.09, 0.05)	0.56	0	0.75
Cholesterol	10,362	−0.0002 (−0.01, 0.01)	−0.03 (−0.10, 0.04)	0.44	0	0.45
HDL cholesterol	10,083	0.01 (−0.0005, 0.02)	−0.06 (−0.16, 0.04)	0.21	39.2	0.13
LDL cholesterol	10,019	0.001 (−0.01, 0.01)	−0.04 (−0.11, 0.03)	0.28	0	0.27
Triglycerides	9257	−0.02 (−0.03, −0.002)	0.09 (−0.05, 0.22)	0.21	64.0	0.13
Glucose	10,321	−0.01 (−0.02, 0.003)	0.03 (−0.04, 0.10)	0.43	0	0.30
BMI	10,377	−0.01 (−0.02, 0.001)	0.01 (−0.07, 0.09)	0.88	15.0	0.72
WHR	10,355	−0.02 (−0.04, −0.01)	−0.02 (−0.09, 0.06)	0.67	0	0.84
Hypertension^4^	8580	0.99 (0.97, 1.01)	0.97 (0.76, 1.24)	0.80	0	0.88

1 All continuous traits are inverse-rank transformed before calculation of *z* score. All effect sizes (95% CIs) are presented in SD units. *I*^2^ is the percentage of total variance in study estimates that is attributable to between-study heterogeneity (29). DBP, diastolic blood pressure; SBP, systolic blood pressure; WHR, waist-hip ratio.

2 All values are means; 95% CIs in parentheses. Values are for observed and expected effect sizes and meta-analysis *P* values.

3 Values are from a random-effects meta-analysis of linear regression coefficients estimated within each study for each phenotype *z* score (on the inverse rank scale) against rs33972313 genotype.

4 Estimates for hypertension were calculated on the log(OR) scale and exponentiated to give an expected OR estimate for hypertension risk.

### Comparison between observed and expected effect estimates

**FIGURE 2:**
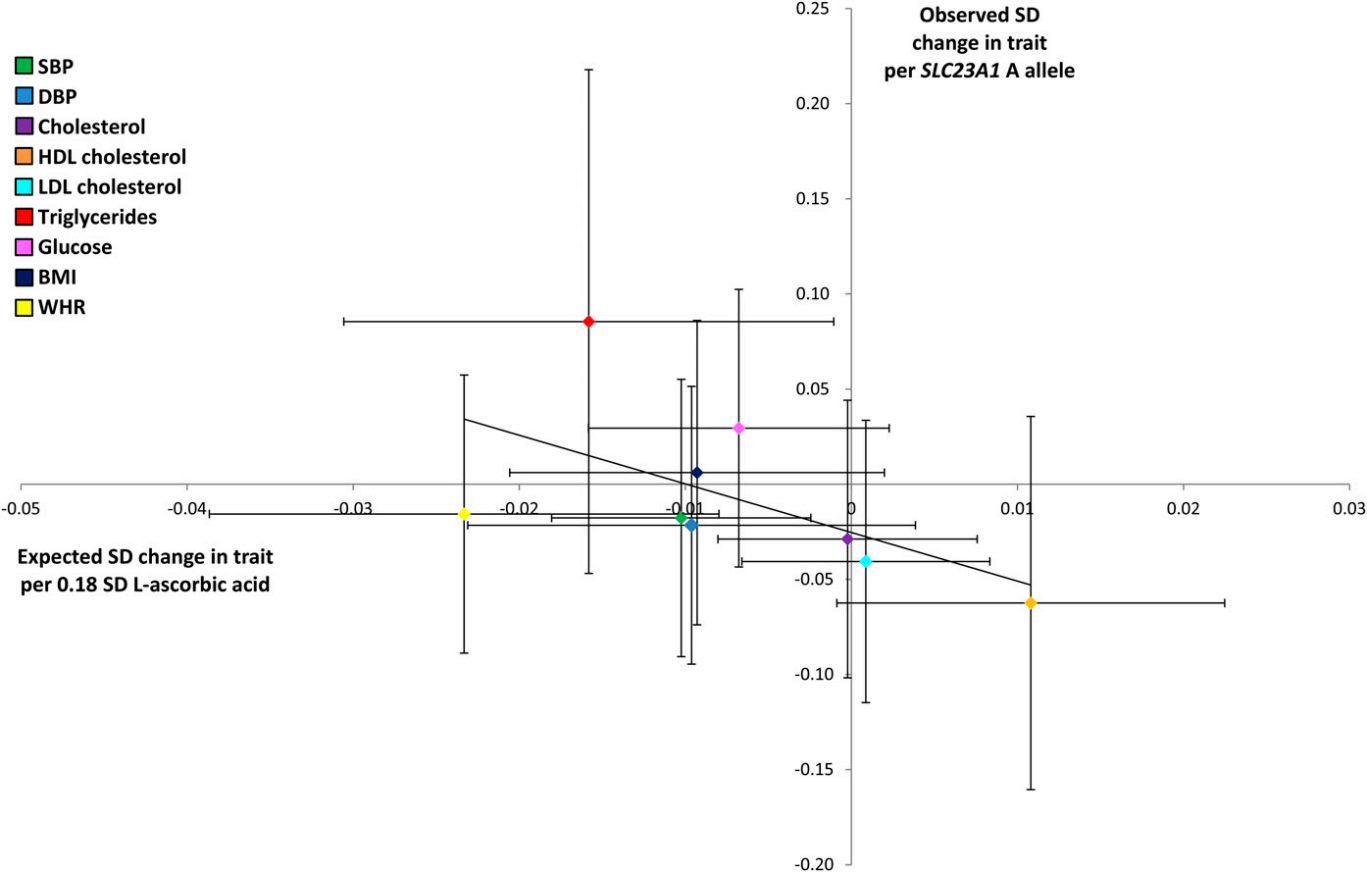
Observed compared with expected effects (95% CIs) of the *SLC23A1* allele on each cardiometabolic trait. Observed effect estimates per *SLC23A1* allele for each cardiometabolic trait are plotted against expected effect estimates given the *SLC23A1*–l-ascorbic acid effect estimate and observed l-ascorbic acid–trait associations. Colors refer to each cardiometabolic trait. DBP, diastolic blood pressure; SBP, systolic blood pressure; *SLC23A1*, solute carrier family 23 member 1; WHR, waist-hip ratio.

The comparison of observed and expected associations between rs33972313 and cardiometabolic traits yielded no substantive evidence of a difference between the 2 sets of results (*P*-comparison values of observed and expected effect estimates ranged between 0.13 and 0.88) (Table 5, **Figure 2**). However, along with the widening of CIs to include the null, observed point estimates for HDL cholesterol, LDL cholesterol, triglycerides, glucose, and BMI were in the opposite direction to those expected (Table 5).

The exclusion of individuals who were known to be on lipid-lowering or antihypertensive medication had no substantive effect on results (**Supplemental Tables S5** and **S6**). Within the EPIC study, results were consistent (Supplemental Table S3), and the inclusion of the EPIC study within the meta-analysis also showed consistent patterns but inflated the level of heterogeneity (**Supplemental Table S7**, **Supplemental Figure S9**).

## DISCUSSION

This study attempted to exploit the association between the *SLC23A1* and l-ascorbic acid to provide a more-reliable estimate of the causal relation between l-ascorbic acid and cardiometabolic health without the limitations present in observational analyses, including reverse causation and confounding.

The observational examination of the relation between l-ascorbic acid concentrations and a collection of cardiometabolic outcomes showed inverse associations between SBP, triglycerides, and the WHR and a positive association with HDL cholesterol. Despite these results, although comparisons between observed and expected effect estimates for the impact of l-ascorbic acid were limited by power, estimates were not suggestive of a causal effect. Taken together, when observed and expected associations between l-ascorbic acid and cardiometabolic outcomes across all traits were compared, there was no suggestion of trend, and if anything, point estimates appeared discordant. Although results were not definitive, in the context of existing work (34), these findings add to the growing evidence suggesting that l-ascorbic acid is an unlikely causal factor in the improvement of cardiometabolic health and suggest that observational associations may be explained by confounding or reverse causation (34, 35).

As to why observational estimates may indicate a beneficial effect of l-ascorbic acid, L-ascorbic acid may be a marker of particular foods and lifestyles, which are genuine risk factors for disease (18, 20, 35, 36). For example, individuals who are more physically active and smoke less may also have high concentrations of circulating l-ascorbic acid as a result or indicator of the healthy lifestyle they lead, which itself is beneficial to cardiometabolic health, as opposed to l-ascorbic acid directly. To this end, these results are in agreement with those of a recent meta-analysis of 20 RCTs (*n* = 294,478), which gave no evidence to support that antioxidant vitamin supplementation has an effect on the incidence of major cardiometabolic events (37).

There were a number of limitations to the current investigation. There was a high level of heterogeneity in the meta-analysis of observational results, which was likely attributable to differences between study protocols and effect estimates. This outcome was indicative of the complications encountered when trying to formulate reliable observational estimates and is in stark contrast to the consistency of genetic data and relations between variation at *SLC23A1* and l-ascorbic acid. This result also highlights the need for alternative approaches, such as the method used in the current study, which lever value out of genetic data in applied epidemiologic analyses to obtain unconfounded and unbiased causal estimates, which seek to add to the weight of evidence. Furthermore, the use of discovery samples in an applied Mendelian randomization (MR) analysis can potentially yield overfitting (38).

Exploratory IV analyses gave first-stage *F* statistics that were either marginal or did not greatly exceed an acceptable threshold to justify the appropriate application of this method for the generation of MR estimates (31). Therefore, it would have been inappropriate to perform an IV analysis in this context because of the use of the collection used in the current study in the discovery of the SNP–l-ascorbic acid relation (23). As the frequency of each minor allele of rs33972313 was ∼4% within the current sample, there was also limited statistical power. Although the observed effect of rs33972313 on circulating concentrations of l-ascorbic acid was relatively large, the rareness of this variant effectively limited the population-based variance explained in l-ascorbic acid and, thus, impeded our ability to draw firm causal inference [as shown elsewhere (39)]. Despite this, rs33972313 remains the best available instrument for the application of this type of analysis.

In conclusion, in the absence of appropriate conditions to undertake a formal MR analysis, the application of the triangulation approach by using a genetic proxy for circulating concentrations of l-ascorbic acid was unable to provide definitive evidence to clarify the causal role of l-ascorbic acid on cardiometabolic health. Results suggest that a larger homogenous study of the impact of circulating l-ascorbic acid should be undertaken by using this design. In the absence of such a study, the current results add to the growing evidence against a likely beneficial role for l-ascorbic acid supplementation in otherwise healthy individuals.

## Supplementary Material

Supplemental data

## Footnotes

5 Abbreviations used: CVD, cardiovascular disease; DBP, diastolic blood pressure; EPIC, European Prospective Investigation into Cancer; IV, instrumental variable; MAF, minor allele frequency; MR, Mendelian randomization; RCT, randomized controlled trial; SBP, systolic blood pressure; *SLC23A1*, solute carrier family 23 member 1; SNP, single nucleotide polymorphism; WHR, waist-hip ratio.
