# Supplementary material for: Variation in the SLC23A1 gene does not influence cardiometabolic outcomes to the extent expected given its association with l-ascorbic acid1
Source: Am J Clin Nutr. 2014 Nov 19;101(1):202–9. doi: 10.3945/ajcn.114.092981 (PMC4266888; doi:10.3945/ajcn.114.092981)
Supplement: Supplemental data [file supp_101_1_202__index.html]

Supplemental data 

# Variation in the *SLC23A1* gene does not influence cardiometabolic outcomes to the extent expected given its association with l-ascorbic acid

## Supplemental data

**Files in this Data Supplement:**

- Supplemental data - Material, Tables 1-7, and Figures 1-9
