# Supplementary material for: Variation in the SLC23A1 gene does not influence cardiometabolic outcomes to the extent expected given its association with l-ascorbic acid1
Source: Am J Clin Nutr. 2014 Nov 19;101(1):202–9. doi: 10.3945/ajcn.114.092981 (PMC4266888; doi:10.3945/ajcn.114.092981)
Supplement: Supplemental data [file 114.092981_ajcn092981SupplementaryData1.docx]

**Supplemental data for the paper entitled “Variation in the *SLC23A1* gene does not influence cardiometabolic outcomes to the extent expected given its association with L-ascorbic acid”**

Kaitlin H. Wade, Nita G. Forouhi, Derek G. Cook, Paul Johnson, Alex McConnachie, Richard W. Morris, Santiago Rodriguez, Zheng Ye, Shah Ebrahim, Sandosh Padmanabhan, Graham Watt, K. Richard Bruckdorfer, Nick J. Wareham, Peter H. Whincup, Stephen Chanock, Naveed Sattar, Debbie A. Lawlor, George Davey Smith and Nicholas J. Timpson

**Corresponding Author:**

Nicholas J. Timpson

MRC Integrative Epidemiology Unit

School of Social and Community Medicine

Oakfield House

Oakfield Grove

Bristol

UK

BS8 2BN

**Email:** [**n.j.timpson@bristol.ac.uk**](mailto:n.j.timpson@bristol.ac.uk)

**Telephone:** 01173310131

**Fax:** 01173310123

**Supplemental Material**

1. Description of study participants and variables used
2. Comparing observed and expected effect estimates

**Supplemental Tables**

**Supplemental Table S1:** Associations of L-ascorbic acid with cardiometabolic outcomes in the EPIC study

**Supplemental Table S2:** Associations between L-ascorbic acid and confounders in

1. BWHHS
2. EPIC
3. MIDSPAN
4. Ten Towns
5. BRHS

**Supplemental Table S3:** Associations of cardiometabolic outcomes with *SLC23A1* variant rs33972313 and with L-ascorbic acid in the EPIC study

**Supplemental Table S4:** Associations between SNP (rs33972313) and confounders in

1. BWHHS
2. EPIC
3. MIDSPAN
4. Ten Towns
5. BRHS

**Supplemental Table S5:** Meta-analysis of associations of L-ascorbic acid with cardiometabolic outcomes (excluding those with lipid-lowering and antihypertensive medication and the EPIC study)

**Supplemental Table S6:** Meta-analysis of associations of cardiometabolic outcomes with *SLC23A1* variant rs33972313 and with L-ascorbic acid (excluding those with lipid-lowering and antihypertensive medication and the EPIC study)

**Supplemental Table S7:** Meta-analysis of associations of cardiometabolic outcomes with *SLC23A1* variant rs33972313 and with L-ascorbic acid (including the EPIC study)

**Supplemental Figures**

**Supplemental Figure S1:** Meta-analysis of the observational per-allele-effect of rs33972313 on L-ascorbic acid including the EPIC study

**Supplemental Figure S2:** Meta-analysis of observational per-allele-effect of rs33972313 on L-ascorbic acid

**Supplemental Figure S3:** Meta-analysis plot for key quantitative traits associated with L-ascorbic acid. Effect sizes (ES) are SD change in trait per 1-SD increase in L-ascorbic acid

**Supplemental Figure S4:** Associations between L-ascorbic acid and

1. SBP
2. DBP
3. Cholesterol
4. HDL cholesterol
5. LDL cholesterol
6. Triglycerides
7. Glucose
8. BMI
9. WHR

**Supplemental Figure S5:** Meta-analysis plot of key quantitative traits associated with L-ascorbic acid. Effect sizes (ES) are SD change in trait per 1-SD increase in L-ascorbic acid (including the EPIC study)

**Supplemental Figure S6:** Meta-analysis plot for key quantitative traits associated with *SLC23A1*. Effect sizes (ES) are SD change in trait per L-ascorbic acid increasing *SLC23A1* allele

**Supplemental Figure S7:** Associations between rs33972313 and

1. SBP
2. DBP
3. Cholesterol
4. HDL cholesterol
5. LDL cholesterol
6. Triglycerides
7. Glucose
8. BMI
9. WHR

**Supplemental Figure S8:** Meta-analysis plot of key quantitative traits associated with *SLC23A1*. Effect sizes (ES) are SD change in trait per L-ascorbic acid increasing *SLC23A1* allele (including the EPIC study)

**Supplemental Figure S9:** Observed effect estimates per *SLC23A1* allele for each cardiometabolic trait including the EPIC study, plotted against expected effect estimates, given the *SLC23A1*-L-ascorbic acid effect estimate and the observed L-ascorbic acid-trait associations. Error bars represent 95% CIs

**Supplemental Material**

***1) Study Participants and Variables Used***

Here, we provide full descriptions of each study used, genotyping, measurements of L-ascorbic acid and cardiometabolic outcomes, and additional covariables used. In all studies, obesity was defined as BMI ≥30kg/m^2^ (weight/height^2^), waist-hip ratio was calculated by dividing waist by hip measurements and smoking status was a (yes/no) binary variable, where “no” was defined as an ex- or non-smoker.

*British Women’s Heart and Health Study (BWHHS)*

Between 1999 and 2001, British women aged 59-80 were randomly selected from 23 British towns and recruited to this prospective cohort study. Participants were interviewed, examined and completed medical questionnaires at baseline. Blood samples, which were taken after a 6-h (minimum) fast and treated with metaphosphoric acid, were used to assess circulating L-ascorbic acid concentration (which was assayed twice and the mean of 2 values was used; *see* Supplemental Data described previously [[1](#_ENREF_1)]), cholesterol, triglycerides, HDL cholesterol, LDL cholesterol and glucose. SNPs were genotyped using the KASPar chemistry and all genotyping was performed by KBiosciences (<http://www.kbioscience.co.uk>). Three stages of internal quality control were used during genotyping. Known locations of non-DNA test controls were used to ensure unique plate identity, a small sample of duplicate DNAs were genotyped for all SNPS, and initial assay validations were performed on a subsample of 96 chromosomes before genotyping the whole sample. A Dinamap 1846SX vital sign monitor was used to measure BP with correction for systematic over-estimation. Measurements were taken twice in succession with a one-minute interval, with the participant seated, rested and their arm supported at chest level. Weight was measured on the Soehnle scales, where a lower mark was recorded if a weight registered between two 0.1kg marks. Standing height was measured after expiration with both hands by the participant’s side. Waist and hip circumference were measured after expiration with the participant standing. Available covariates included age, insulin, C-reactive protein (CRP), interleukin-6 (Il-6) and adiponectin, smoking status, aspirin and statin use, alcohol intake, physical activity, socioeconomic position and obesity. Aspirin usage and statin usage were dichotomized into (yes/no) binary variables. Alcohol intake was categorised into drinking either “most days”, “at weekends”, “once or twice a month”, “on special occasions” or “never”. Physical activity was categorised as exercising “less than 2 hours”, “between 2 and 3 hours” or “more than 3 hours” per week. Socioeconomic position including information on 10 variables: whether the participant’s child had no car, no bathroom, no hot water, shared bedroom, no household car, council accommodation, state pension, and minimal education, and whether the participant or the participant’s child was of a manual status. Those with zero or 10 of these variables were excluded due to low numbers; therefore, the socioeconomic position variable was defined as a 9-group categorical variable describing how many of these 10 variables the participant had. Data was available from 4,286 individuals for this study, of which 1,833 had full data on genotypes, L-ascorbic acid and cardiometabolic outcomes available.

*The European Prospective Investigation into Cancer Norfolk study (EPIC Norfolk)*

Data from 7,563 individuals from the Norfolk arm of the European Prospective Investigation into Cancer and Nutrition (EPIC-Norfolk) were analysed, of whom 3,737 had full data available on genotypes, L-ascorbic acid and cardiometabolic outcomes. This is a prospective study of men and women in Norfolk, United Kingdom, aged between 40 and 79 years at recruitment (1993 - 1997). Plasma L-ascorbic acid concentration was measured among samples stabilised with metaphosphoric acid and stored at -70C and estimated using a fluorometric assay within one week of sampling [[2](#_ENREF_2)]. Genotyping of the SNPs using Custom Taqman assays (Applied Biosystems, Warrington, UK) on an ABI PRISM 7900HT Sequence Detection System. The genotyped SNPs had a genotyping call rate of ≥96.8%. BP was measured using an Accutorr Sphygmomanometer (Datascope, UK), after the participant was seated for 3 minutes, with the arm held horizontal at the level of the mid-sternum. A total of 42ml of blood was collected in EDTA, citrated and plain Monovettes. The citrated and plain blood samples were kept overnight in a refrigerator, and the EDTA samples at room temperature. Height was measured to the nearest millimetre, without shoes using a free-standing stadiometer, and weight was measured to the nearest 0.2kg, without shoes in light clothing using digital scales (Salter, UK). Waist was measured using the definition as either minimum circumference at the natural waistline between lower rib margin and the iliac crest (with belts removed) or, when the minimum circumference was not identifiable, as the circumference at the navel level. Hip circumference was the maximum measurement between the iliac crest and the crotch, where the greatest of 2 measurements was used. Total cholesterol, HDL cholesterol and triglycerides were measured on the RA 1000 equipment (Bayer Diagnostics, Basingstoke) and LDL cholesterol was calculated by using the Friedewald formula (Friedewald *et al*., 1972) except where triglyceride level was >4mmol^-1^. Available covariates included age, CRP, alcohol intake, physical activity, current smoking status, obesity, social class, education and usage of lipid-lowering and hypertension medications. Alcohol intake was classified as drinking 0, 1-7, 7-21, 21-70, 70+ units per week. Physical activity was categorised into “inactive”, “moderately inactive”, “moderately active” or “active”. Social class was categorised into “professor”, “managerial”, “non-manually skilled”, “manually skilled”, “part skilled” or “unskilled”. Education was categorised into having no qualifications, O-levels, A-levels or a Degree. Both the EPIC study and the BRHS had records of both lipid-lowering and antihypertensive medications, which were (yes/no) binary variables.

*MIDSPAN*

This is a large-scale occupational cohort study based in Scotland consisting of 4 separate occupational and general population cohort studies. The three original studies took place between 1964 and 1976, recruiting from 27 factories and workplaces from central Scotland. In 1996, the next generation was studied when offspring of couples in the original group from Paisley and Renfrew were recruited into the family study. The recent study in 1996 included 1,040 sons and 1,298 daughters aged 30-59 from 1,477 families (a total of 2,338 participants). Families were removed to remove non-independent observations, leaving 1,477 non-related participants. L-ascorbic acid status was measured using HPLC with electrochemical detection in blood samples treated with metaphosphoric acid at the point of collection. Genotyping was performed on a ABI PRISM 7900HT sequence detection system using a Taqman assay developed by Applied Biosystems followed by allelic discrimination using Applied Biosystems software [[3](#_ENREF_3), [4](#_ENREF_4)]. All genotyping errors were manually resolved by checking raw genotype data, where individuals were either blanked (zeroed) or corrected prior to analysis. Average BP was taken using the automated Dinamapp 8100 instrument with the subject seated after five minutes of rest, where three BP readings were taken and the average of the last two readings was used [[5](#_ENREF_5)]. Measurements of height, weight, hip and waist were obtained by a qualified research nurse who also collected non-fasting venuous blood samples, from which plasma was separated and aliquoted and stored at -80⁰C for subsequent analysis of cholesterol (LDL, HDL and total), glucose and triglycerides[[6](#_ENREF_6)]. Available covariates included age, sex, urate, smoking status, and prevalence of diabetes. The prevalence of diabetes was described as a (yes/no) binary variable. Full data on genotypes, L-ascorbic acid and cardiometabolic metabolic outcomes were available for 1,138 participants.

*Ten Towns*

This is a longitudinal study of 1,531 children and adolescents between the ages of 12 and 16 from 10 British towns. The third phase of this study took place in 72 secondary schools across England and Wales in 1998-2000, where whole blood and plasma samples were provided and DNA was later extracted and L-ascorbic acid measured. L-ascorbic acid was measured in plasma treated with metaphosphoric acid at the point of collection and estimated using a fluorimetric assay. Genotyping and quality control was carried out by KBiosciences using essentially identical methods to those used in the BWHHS. BP was measured after five minutes of rest, where two measures were taken at one-minute intervals using the Dinamap 1846SX instrument and an appropriately sized cuff selected on the basis of arm circumference and in accordance with the AHA recommendations [[7](#_ENREF_7)]. Details of how height, weight, waist, hip and glucose levels have been described previously [[8](#_ENREF_8), [9](#_ENREF_9)]. All participants were examined in light clothing and no shoes. Height was measured to the last complete millimetre with a portable stadiometer (CMS Ltd, Camden, UK), and weight to the last complete 0.1kg with a digital electronic weighing scale (Soehnle Ltd, Murrhardt, Germany). Waist circumference was measured at the end of normal expiration at the mid-point between the iliac crest and the lower edge of the ribs in the mid-axillary line, with the child standing with feet 15cm apart, and hip circumference was measured at the point of maximum circumference over the buttocks. A venous blood sample was collected after an overnight fast, separated and frozen at -20⁰C within 6 hours of collection and transferred to central laboratories for analysis. Plasma glucose was measured in a fluoride-oxalate sample with a Falcor 600 automated analyser, and cholesterol concentration was measured with the Technicon Dax system (method No SM4-2143F90). Available covariates included age, sex, insulin CRP, alcohol intake, physical activity, physical comparison, smoking status, social class and obesity. Alcohol intake was categorised as drinking “very frequently”, “not often” and “never”. Physical activity was categorised as exercising “very often”, “fairly little”, and “not often” during a week and physical comparison was a variable describing whether the individual felt they were “more”, “average” or “less” active than peers of the same age and sex. Social class was an 8-item categorical group: I, II, IIINM, IV, V, IIIM, and two unclassified categories. Full data on genotypes, L-ascorbic acid and cardiometabolic outcomes for 1,324 participants was available.

*British Regional Heart Study (BRHS)*

Initiating in 1978, this is a prospective study in men aged between 40 and 59 years drawn from general practices in 24 British towns. A total of 7,735 men were initially recruited and 4,252 men (55% response rate, 77% of those still alive) were re-examined in 1998-2000. At the time of the current study, 3,945 participants were included, of whom 2,521 had full data on genotypes, L-ascorbic acid and cardiometabolic outcomes. Measurements of L-ascorbic acid, genotyping of SNPs and genotype quality control were carried out in the same laboratories, using the same methods as for the BWHHS. BP was measured twice in the right arm using the Dinamapp 1846SX oscillometric recorder, where the average of these readings was used [[10](#_ENREF_10)]. Subjects had measurements of height, weight and waist-hip ratio. Details of how blood samples were obtained in non-fasting state for biochemical analysis of lipids have been described previously [[11](#_ENREF_11), [12](#_ENREF_12)]. Total cholesterol, HDL cholesterol, LDL cholesterol and triglycerides were measured on a Hitachi 747 automated analyzer using established methods [[13](#_ENREF_13)]. Height was measured without shoes using a Harpenden Stadiometer to the nearest millimetre, and weight was taken in trousers and socks on an MPS110 field survey scale to the nearest 0.1kg [[12](#_ENREF_12)]. Waist circumference was taken from the midpoint between the iliac crest and the lower ribs measured at the side, and hip circumference was measured at the point of maximum circumference over the buttocks [[14](#_ENREF_14)]. Available covariates included age, insulin, CRP, Il-6 and urate, use of lipid lowering medication and aspirin, smoking status, alcohol intake, physical activity, social class and obesity. Aspirin usage was a (yes/no) binary variable describing current usage. Alcohol intake was separated into “non-“, “occasional” or a “light”, “moderate” or “heavy” drinker. Weekly physical activity was categorised into “inactive”, “occasional activity”, “light”, “moderate”, “moderately vigorous” or “vigorous” activity per week. Social class was either manual or non-manual.

***Ethical Approval***

The appropriate Local Ethics Committees gave permission for data collection from the BWHHS, the MIDSPAN study and the Ten Towns study, and UK multicentre ethical approvals were obtained for the BRHS. Genetic testing was approved by the Welsh Research Ethics Committee for the Ten Towns study. Participants in the BWHHS, the BRHS, the EPIC study and the MIDSPAN study were additionally asked for informed consent to review medical records and for permission to perform anonymised genetic testing of stored blood. Only individuals who gave appropriate consent were included within current analysis.

***2) Comparing observed and expected effect estimates***

Firstly, the standard errors (SEs) of expected effect sizes were calculated using the Taylor series expansion of the product of the observed and expected estimates. These were used to generate the corresponding 95% confidence intervals for the expected effect estimates. Then, to assess any difference in observed and expected effect estimates of the association between *SLC23A1*-outcome, we computed a Z-statistic using the following formula:

$$Z=\frac{\left[ \beta_{1}- \beta_{2} \right]}{SE[\beta_{1}- \beta_{2}]}$$

where $\beta_{1}$ and $\beta_{2}$ are the observed and expected effect estimates, and $SE[\beta_{1}- \beta_{2}]$ is the standard error of the difference in effect sizes. The denominator of this equation was calculated in the following way.

$$SE\left[ \beta_{1}- \beta_{2} \right]= \sqrt{{\{(SE[\beta_{1}])}^{2}+{(SE[\beta_{2}])}^{2}- 2Cov[\beta_{1},\beta_{2}]\}}$$

where $Cov[\beta_{1},\beta_{2}]$ is the covariance between $\beta_{1}$ and $\beta_{2}$. The obtained z-statistic was then compared against the normal distribution to generate a two-tailed *P*-value for comparing the observed and expected estimates.

**Supplemental Tables**

**Supplemental Table S1:** Associations of L-ascorbic acid with cardiometabolic outcomes in the EPIC study

| **Phenotype** | **N** | **Observed change in outcome per SD change in L-ascorbic acid** | ***P*-value^1^** |
| --- | --- | --- | --- |
| SBP | 7,096 | -0.14  (-0.17, -0.12) | 2.01x10^-33^ |
| DBP | 7,096 | -0.13  (-0.15, -0.11) | 1.34x10^-28^ |
| Cholesterol | 7,039 | -0.02  (-0.05, -0.001) | 0.04 |
| HDL cholesterol | 6,746 | 0.22  (0.20, 0.25) | 1.31x10^-77^ |
| LDL cholesterol | 6,680 | -0.04  (-0.06, -0.02) | 0.001 |
| Triglycerides | 7,039 | -0.22  (-0.24, -0.20) | 6.71x10^-79^ |
| Glucose | 5,716 | -0.10  (-0.13, -0.08) | 3.32x10^-15^ |
| BMI | 7,106 | -0.17  (-0.19, -0.15) | 2.49x10^-47^ |
| WHR | 7,100 | -0.32  (-0.34, -0.29) | 2.49x10^-169^ |
| Hypertension^2^ | 5,699 | 0.75  (0.71, 0.80) | 2.48x10^-21^ |

^1^ *P-values are from linear regression coefficients estimated within the EPIC study for each phenotype Z-score (on the inverse rank scale) against L-ascorbic acid Z-score (inverse rank scale).*

*^2^ Hypertension was defined as a systolic blood pressure ≥140mmHg and a diastolic blood pressure ≥90mmHg*

**Supplemental Table S2: Associations between L-ascorbic acid and confounders in (A) BWHHS, (B) EPIC, (C) MIDSPAN, (D) Ten Towns and (E) BRHS**

**Supplemental Table S2A:** Association between L-ascorbic acid levels and confounders in the BWHHS

| **Variable** | **N** | **β-coefficients^1^ (95% CI)** | ***P*-value** |
| --- | --- | --- | --- |
| Age (years)^2^ | 3,606 | 0.01  (-0.2, 0.2) | 0.94 |
| Insulin (log pmol/l)^3^ | 3,581 | -5.64  (-7.01, -4.27) | 9.85x10^-16^ |
| C-Reactive Protein (log mg/L)^3^ | 3,475 | -3.10  (-3.94, -2.26) | 6.71x10^-13^ |
| Il-6 (log pg/mL)^3^ | 3,584 | -5.04  (-6.40, -3.69) | 3.52x10^-13^ |
| Adiponectin (µg/mL) | 457 | -0.01  (-0.3, 0.3) | 0.95 |
| Smoking status^4^ | 3,604 | -12.20  (-15.11, -9.28) | 3.36x10^-16^ |
| Aspirin Usage^5^ | 3,597 | -1.81  (-4.49, 0.87) | 0.19 |
| Statin Usage^5^ | 3,597 | -2.58  (-6.11, 0.94) | 0.15 |
| Alcohol Intake^6^ | 2,734 | -2.51  (-3.26, -1.77) | 3.90x10^-11^ |
| Physical Activity^7^ | 2,985 | 1.81  (0.54, 3.07) | 0.01 |
| Obesity^8^ | 3,573 | -5.36  (-7.44, -3.27) | 4.92x10^-7^ |
| Socioeconomic Position^9^ | 2,977 | -2.21  (-2.65, -1.77) | 9.23x10^-23^ |

^1^ *All regression coefficients are adjusted for age, where β-coefficients represent average change in L-ascorbic acid for each unit increase in every continuous or categorical variable*

^2^ *Not adjusted for age*

^3^ *Insulin, c-reactive protein and il-6 were log-transformed*

^4^ *Smoking status was a (yes/no) binary variable describing current smoking status*

^5^ *Aspirin and statin usage were (yes/no) binary variables describing current usage*

^6^ *Alcohol intake was categorised as drinking ‘most days’, ‘at weekends’, ‘once or twice a month’, ‘on special occasions’ and ‘never’.*

^7^ *Physical activity was categorised as exercising ‘less than 2 hours’, ‘between 2 and 3 hours’ and ‘more than 3 hours’ a week*

^8^ *Obesity was classified as having a BMI ≥30kg/m^2^*

*^9^ Socioeconomic position was classified as the total sum of variables indicative of a low socio-economic position*

**Supplemental Table S2B:** Association between L-ascorbic acid levels and confounders in the EPIC study

| **Variable** | **N** | **β-coefficients^1^ (95% CI)** | ***P*-value** |
| --- | --- | --- | --- |
| Age (years)^2^ | 7,106 | -0.13  (-0.18, -0.08) | 5.44x10^-7^ |
| C-Reactive Protein (log mg/L)^3^ | 5,652 | -4.00  (-4.49, -3.52) | 6.68x10^-59^ |
| Alcohol Intake^4^ | 7,046 | 1.92  (1.31, 2.53) | 6.41x10^-10^ |
| Physical activity^5^ | 7,106 | 2.16  (1.56, 2.76) | 1.67x10^-12^ |
| Current Smoking Status^6^ | 7,053 | -4.63  (-5.32, -3.95) | 1.55x10^-40^ |
| Obesity Prevalence^7^ | 7,106 | -6.17  (-7.42, -4.93) | 2.73x10^-22^ |
| Social Class^8^ | 6,948 | -1.94  (-2.28, -1.59) | 5.95x10^-28^ |
| Education^9^ | 7,105 | 2.50  (2.07 2.93) | 2.10x10^-30^ |
| Lipid-lowering medication^10^ | 7,106 | -1.40  (-5.02, 2.21) | 0.45 |
| Antihypertension medication^11^ | 7,106 | -2.67  (-3.86, -1.48) | 1.03x10^-5^ |

^1^ *All regression coefficients are adjusted for age and sex, where β-coefficients represent average change in L-ascorbic acid for each unit increase in every continuous or categorical variable*

^2^ *Not adjusted for age*

^3^ *C-reactive protein was log-transformed*

^4^ *Alcohol intake was categorised by 0, 1-7, 7-21, 21-70, 70+ units per week*

^5^ *Physical activity was categorised into inactive, moderately inactive, moderately active or active*

^6^ *Smoking status was categorised as never, former or current*

^7^ *Obesity was defined as BMI ≥30kg/m^2^*

^8^ *Social class was categorised into professor, managerial, non-manually skilled, manually skilled, part skilled or unskilled*

^9^ *Education was categorised into no qualifications, O-levels, A-levels or Degree*

^10^ *Lipid-lowering medication was a yes/no binary variable*

^11^ *Antihypertensive medication was a yes/no binary variable*

**Supplemental Table S2C:** Association between L-ascorbic acid levels and confounders in the MIDSPAN study

| **Variable** | **N** | **β-coefficients^1^ (95% CI)** | ***P*-value** |
| --- | --- | --- | --- |
| Age (years)^2^ | 1,364 | -0.10  (-0.32, 0.12) | 0.36 |
| Urate (µmol/L) | 1,345 | -0.02  (-0.04, -0.004) | 0.02 |
| Smoking status^3^ | 1,364 | -8.52  (-10.10, -6.94) | 3.24x10^-25^ |
| Obesity^4^ | 1,362 | -8.47  (-11.95, -5.00) | 1.85x10^-6^ |
| Prevalence of diabetes | 1,364 | 1.84  (-8.69, 12.37) | 0.73 |

^1^ *All regression coefficients are adjusted for age and sex, where β-coefficients represent average change L-ascorbic acid for each unit increase in every continuous or categorical variable*

^2^ *Not adjusted for age*

^3^ *Smoking status was categorised as ‘never’, ‘former’ and ‘current’ smokers*

^4^ *Obesity was classified as having a BMI ≥30kg/m^2^*

**Supplemental Table S2D:** Association between L-ascorbic acid levels and confounders in the Ten Towns study

| **Variable** | **N** | **β-coefficients^1^ (95% CI)** | ***P*-value** |
| --- | --- | --- | --- |
| Age (years)^2^ | 1,531 | 0.05  (-2.01, 2.10) | 0.96 |
| Insulin (log pmol/l)^3^ | 1,520 | -0.74  (-3.29, 1.80) | 0.57 |
| C-Reactive Protein (log mg/L)^3^ | 1,524 | 0.12  (-0.86, 1.11) | 0.81 |
| Alcohol Intake^4^ | 1,432 | -2.42  (-4.49, -0.34) | 0.02 |
| Physical Activity^5^ | 1,473 | -4.48  (-6.56, -2.20) | 8.59x10^-5^ |
| Physical Comparison^6^ | 1,515 | -2.15  (-3.80, -0.50) | 0.01 |
| Smoking status^7^ | 1,524 | 0.47  (-1.03, 1.97) | 0.54 |
| Social Class^8^ | 1,474 | -1.43  (-2.06, -0.80) | 8.60x10^-6^ |
| Obesity^9^ | 1,525 | 1.54  (-5.30, 8.39) | 0.66 |

^1^ *All regression coefficients are adjusted for age and sex, where β-coefficients represent average change in L-ascorbic acid for each unit increase in every continuous or categorical variable*

^2^ *Not adjusted for age*

^3^ *Insulin and c-reactive protein were log-transformed*

^4^ *Alcohol intake was categorised as drinking ‘very frequently’, ‘not often’ and ‘never’*

^5^ *Physical activity was categorised as exercising ‘very often’, ‘fairly little’ and ‘not often’*

^6^ *Physical comparison was categorised as being ‘more’, ‘average’ or ‘less’ active than peers of same age and sex*

^7^ *Smoking status was categorised as ‘never’, ‘former’ and ‘current’ smokers*

^8^ *Social class was an 8-item categorical group ranging from social class ‘I’, ‘non-manual’, ‘manual’ and ‘unclassified’*

^9^ *Obesity was classified as having a BMI ≥30kg/m^2^*

**Supplemental Table S2E:** Association between L-ascorbic acid levels and confounders in the BRHS

| **Variable** | **N** | **β-coefficients^1^ (95% CI)** | ***P*-value** |
| --- | --- | --- | --- |
| Age (years)^2^ | 3,811 | 0.03  (-0.1, 0.2) | 0.75 |
| Insulin (log pmol/L)^3^ | 3,766 | -1.69  (-3.03, -0.35) | 0.01 |
| C-Reactive Protein (log mg/L)^3^ | 3,802 | -1.62  (-2.39, -0.85) | 3.96x10^-5^ |
| Il-6 (log pg/mL)^3^ | 2,206 | -1.13  (-2.61, 0.35) | 0.14 |
| Urate (µmol/L) | 3,777 | -11.31  (-22.04, -0.58) | 0.04 |
| Lipids lowering medication^4^ | 3,811 | 2.82  (-0.52, 6.16) | 0.10 |
| Smoking status^5^ | 3,805 | -6.77  (-9.38, -4.16) | 3.58x10^-7^ |
| Aspirin intake^4^ | 3,707 | -0.92  (-2.98, 1.14) | 0.38 |
| Alcohol Intake^6^ | 3,735 | -0.61  (-1.94, 0.72) | 0.37 |
| Physical Activity^7^ | 3,674 | 1.62  (0.60, 2.63) | 0.002 |
| Social class^8^ | 3,710 | -4.54  (-6.32, -2.75) | 6.36x10^-7^ |
| Obesity^9^ | 3,794 | -2.41  (-4.75, -0.06) | 0.04 |

^1^ *All regression coefficients are adjusted for age and sex, where β-coefficients represent average change in L-ascorbic acid for each unit increase in every continuous or categorical variable*

^2^ *Not adjusted for age*

^3^ *Insulin, c-reactive protein and il-6 were log-transformed*

^4^ *Aspirin usage was a (yes/no) binary variable describing current usage*

^5^ *Smoking status was a (yes/no) binary variable describing current smoking status*

^6^ *Alcohol intake was categorised as a non-, occasional, or a light/moderate/heavy drinker.*

^7^ *Physical activity was categorised as inactive or occasional activity, light, moderate, or moderately vigorous or vigorous*

^8^ *Social class was classified as non-manual and manual*

^9^ *Obesity was classified as having a BMI ≥30kg/m^2^*

**Supplemental Table S3:** Associations of cardiometabolic outcomes with *SLC23A1* variant rs33972313 and with L-ascorbic acid in the EPIC study

| **Phenotype** | ***N*** | **Expected change in outcome Z-score per effect allele** | **Observed change in outcome Z-score per effect allele** | ***P*-value**  **(rs33972313 vs. outcome)^1^** | ***P*-value for difference between observed and expected** |
| --- | --- | --- | --- | --- | --- |
| SBP | 6,387 | -0.06  (-0.07, -0.04) | 0.04  (-0.05, 0.13) | 0.40 | 0.04 |
| DBP | 6,387 | -0.05  (-0.07, -0.04) | 0.03  (-0.07, 0.12) | 0.59 | 0.10 |
| Cholesterol | 6,203 | -0.01  (-0.02, -0.0001) | 0.01  (-0.09, 0.10) | 0.89 | 0.73 |
| HDL cholesterol | 6,029 | 0.09  (0.07, 0.11) | 0.03  (-0.06, 0.12) | 0.54 | 0.20 |
| LDL cholesterol | 5,982 | -0.02  (-0.03, -0.01) | 0.01  (-0.09, 0.11) | 0.84 | 0.60 |
| Triglycerides | 6,203 | -0.09  (-0.11, -0.07) | -0.02  (-0.11, 0.07) | 0.69 | 0.12 |
| Glucose | 4,940 | -0.04  (-0.06, -0.03) | 0.005  (-0.09, 0.10) | 0.92 | 0.33 |
| BMI | 6,396 | -0.07  (-0.09, -0.05) | -0.02  (-0.11, 0.07) | 0.63 | 0.31 |
| WHR | 6,390 | -0.13  (-0.16, -0.10) | -0.04  (-0.14, 0.05) | 0.33 | 0.07 |
| Hypertension^2^ | 5,194 | 0.89  (0.86, 0.92) | 1.02  (0.75, 1.23) | 0.87 | 0.17 |

*Data are means (95% CI) for observed and expected effect sizes. All continuous traits are inverse-rank transformed before calculation of Z-score. All effect sizes (95% CIs) are presented in SD units.*

*^1^ P-values are from linear regression coefficients estimated within the EPIC study for each phenotype Z-score (on the inverse rank scale) against rs33972313 genotype.*

*^2^ Estimates for hypertension were calculated on the log(OR) scale, then exponentiated to give an expected OR estimate for hypertension risk.*

**Supplemental Table** **S4: Associations between SNP (rs33972313) and confounders in (A) BWHHS, (B) EPIC**, **(C) MIDSPAN, (D) Ten Towns and (E) BRHS**

**Supplemental Table S4A:** Associations between SNP and confounders in the BWHHS (n = 3,718)

| **Variable** | **rs33972313** | |
| --- | --- | --- |
|  | **β-coefficients^1^ (95% CI)** | ***P*-value** |
| Age (years)^2^ | 0.26  (-0.43, 0.95) | 0.46 |
| Insulin (log pmol/l) ^3^ | -0.04  (-0.13, 0.04) | 0.29 |
| C-Reactive Protein (log mg/L)^3^ | -0.07  (-0.21, 0.07) | 0.33 |
| Il-6 (log pg/mL)^3^ | -0.04  (-0.1, 0.04) | 0.32 |
| Adiponectin (µg/mL) | 0.2  (-3.0, 3.4) | 0.90 |
| Smoking status^4^ | 0.77  (0.49, 1.21) | 0.26 |
| Aspirin Usage^5^ | 1.08  (0.76, 1.54) | 0.67 |
| Statin Usage^5^ | 0.93  (0.57, 1.53) | 0.77 |
| Alcohol Intake^6^ | 0.96  (0.66, 1.38) | 0.81 |
| Physical Activity^7^ | 0.96  (0.67, 1.35) | 0.80 |
| Obesity^8^ | 0.91  (0.68, 1.22) | 0.54 |
| Socioeconomic Position^9^ | 0.03  (-0.29, 0.36) | 0.84 |

^1^ *All regression coefficients are adjusted for age, where β-coefficients represent average change in each continuous variable and the OR of each categorical variable with an increase of minor allele frequency of both SNPs*

^2^ *Not adjusted for age*

^3^ *Insulin, c-reactive protein and il-6 were log-transformed*

^4^ *Smoking status was a (yes/no) binary variable describing current smoking status*

^5^ *Aspirin and statin usage were (yes/no) binary variables describing current usage*

^6^ *Alcohol intake was categorised as drinking ‘most days’, ‘at weekends’, ‘once or twice a month’, ‘on special occasions’ and ‘never.*

^7^ *Physical activity was categorised as exercising ‘less than 2 hours’, ‘between 2 and 3 hours’ and ‘more than 3 hours’ a week*

^8^ *Obesity was classified as having a BMI ≥30kg/m^2^*

*^9^ Socioeconomic position was classified as the total sum of variables indicative of a low socio-economic position*

**Supplemental Table S4B:** Associations between SNP and confounders in the EPIC study (n = 6,396)

| **Variable** | **rs33972313** | |
| --- | --- | --- |
|  | **β-coefficients^1^ (95% CI)** | ***P*-value** |
| Age (years)^2^ | -0.14  (-1.00, 0.71) | 0.74 |
| C-Reactive Protein (log mg/L)^3^ | 0.09  (-0.01, 0.20) | 0.09 |
| Alcohol Intake^4^ | 0.95  (0.72, 1.26) | 0.74 |
| Physical activity^5^ | 0.96  (0.75, 1.22) | 0.74 |
| Current Smoking Status^6^ | 0.88  (0.72, 1.06) | 0.18 |
| Obesity Prevalence^7^ | 1.25  (0.97, 1.61) | 0.08 |
| Social Class^8^ | 1.06  (0.73, 1.55) | 0.75 |
| Education^9^ | 1.07  (0.87, 1.31) | 0.53 |
| Lipid-lowering medication^10^ | 1.42  (0.69, 2.89) | 0.34 |
| Hypertension medication^11^ | 1.02  (0.79, 1.33) | 0.85 |

^1^ *All regression coefficients are adjusted for age and sex, where β-coefficients represent average change in L-ascorbic acid for each unit increase in every continuous or categorical variable*

^2^ *Not adjusted for age*

^3^ *C-reactive protein was log-transformed*

^4^ *Alcohol intake was categorised by 0, 1-7, 7-21, 21-70, 70+ units per week*

^5^ *Physical activity was categorised into inactive, moderately inactive, moderately active or active*

^6^ *Smoking status was categorised as never, former or current*

^7^ *Obesity was defined as BMI ≥30 kg/m^2^*

^8^ *Social class was categorised into professor, managerial, non-manually skilled, manually skilled, part skilled or unskilled*

^9^ *Education was categorised into no qualifications, O-levels, A-levels or Degree*

^10^ *Lipid-lowering medication was a yes/no binary variable*

^11^ *Antihypertensive medication was a yes/no binary variable*

**Supplemental Table S4C:** Associations between SNP and confounders in the MIDSPAN study (n = 1,379)

| **Variable** | **rs33972313** | |
| --- | --- | --- |
|  | **β-coefficients^1^ (95% CI)** | ***P*-value** |
| Age (years)^2^ | 0.35  (-0.68, 1.39) | 0.50 |
| Urate (µmol/L) | -4.91  (-16.69, 6.87) | 0.41 |
| Smoking status | 1.14  (0.81, 1.62) | 0.45 |
| Obesity^3^ | 1.02  (0.64, 1.58) | 0.99 |
| Prevalence of diabetes | 1.49  (0.50, 4.52) | 0.47 |

^1^ *All regression coefficients are adjusted for age and sex, where β-coefficients represent average change in each continuous variable and the OR of each categorical variable with an increase in minor allele frequency of both SNPs*

^2^ *Not adjusted for age*

^3^ *Obesity was classified as having a BMI ≥30kg/m^2^*

**Supplemental Table S4D:** Associations between SNP and confounders in the Ten Towns study (n = 1,477)

| **Variable** | **rs33972313** | |
| --- | --- | --- |
|  | **β-coefficients^1^ (95% CI)** | ***P*-value** |
| Age (years)^2^ | -0.08  (-0.19, 0.03) | 0.17 |
| Insulin (log pmol/l)^3^ | 0.04  (-0.05, 0.13) | 0.37 |
| C-Reactive Protein (log mg/L)^3^ | -0.004  (-0.24, 0.23) | 0.97 |
| Alcohol Intake^4^ | 0.95  (0.61, 1.49) | 0.83 |
| Physical Activity^5^ | 2.64  (1.14, 6.08) | 0.02 |
| Physical Comparison^6^ | 1.3  (0.9, 2.1) | 0.17 |
| Smoking status^7^ | 0.80  (0.54, 1.19) | 0.27 |
| Social Class^8^ | 0.99  (0.49, 1.99) | 0.97 |
| Obesity^9^ | 1.24  (0.45, 3.47) | 0.68 |

^1^ *All regression coefficients are adjusted for age and sex, where β-coefficients represent average change in each continuous variable and the OR of each categorical variable with an increase of minor allele frequency of both SNPs*

^2^ *Not adjusted for age*

^3^ *Insulin and c-reactive protein were log-transformed*

^4^ *Alcohol intake was categorised as drinking ‘very frequently’, ‘not often’ and ‘never’*

^5^ *Physical activity was categorised as exercising ‘very often’, ‘fairly little’ and ‘not often’*

^6^ *Physical comparison was categorised as being ‘more’, ‘average’ or ‘less’ active than peers of same age and sex*

^7^ *Smoking status was categorised as ‘never’, ‘former’ and ‘current’ smokers*

^8^ *Social class was an 8-item categorical group ranging from social class ‘I’, ‘non-manual’, ‘manual’ and ‘unclassified’*

^9^ *Obesity was classified as having a BMI ≥30kg/m^2^*

**Supplemental Table S4E:** Associations between SNP and confounders in the BRHS (n = 3,870)

| **Variable** | **rs33972313** | |
| --- | --- | --- |
|  | **β-coefficients^1^ (95% CI)** | ***P*-value** |
| Age (years)^2^ | 0.62  (-0.05, 1.29) | 0.07 |
| Insulin (log pmol/L)^3^ | 0.02  (-0.06, 0.10) | 0.63 |
| C-Reactive Protein (log mg/L)^3^ | 0.08  (-0.06, 0.22) | 0.29 |
| Il-6 (log pg/mL)^3^ | 0.07  (-0.04, 0.17) | 0.19 |
| Urate (µmol/L) | 0.002  (-0.01, 0.01) | 0.74 |
| Lipids lowering medication^4^ | 0.59  (0.33, 1.07) | 0.08 |
| Smoking status^5^ | 1.00  (0.69, 1.45) | 0.99 |
| Aspirin intake^4^ | 1.02  (0.77, 1.36) | 0.88 |
| Alcohol Intake^6^ | 0.74  (0.51, 1.07) | 0.11 |
| Physical Activity^7^ | 1.08  (0.82, 1.41) | 0.59 |
| Social class^8^ | 1.36  (1.06, 1.76) | 0.02 |
| Obesity^9^ | 1.36  (1.01, 1.85) | 0.05 |

^1^ *All regression coefficients are adjusted for age and sex, where β-coefficients represent average change in each continuous variable and the OR of each categorical variable with an increase of minor allele frequency of both SNPs*

^2^ *Not adjusted for age*

^3^ *Insulin, c-reactive protein and il-6 were log-transformed*

^4^ *Aspirin usage was a (yes/no) binary variable describing current usage*

^5^ *Smoking status was a (yes/no) binary variable describing current smoking status*

^6^ *Alcohol intake was categorised as a non-, occasional, or a light/moderate/heavy drinker.*

^7^ *Physical activity was categorised as inactive or occasional activity, light, moderate, or moderately vigorous or vigorous*

^8^ *Social class was classified as non-manual and manual*

^9^ *Obesity was classified as having a BMI ≥30kg/m^2^*

**Supplemental Table S5:** Meta-analysis of associations of L-ascorbic acid with cardiometabolic outcomes (excluding those with lipid-lowering and antihypertensive medication and the EPIC study)

| **Phenotype** | **N** | **Observed change in outcome per SD change in L-ascorbic acid** | ***P*-value^1^** | ***I^2^ (%)*** |
| --- | --- | --- | --- | --- |
| SBP | 8,462 | -0.05  (-0.10, -0.01) | 0.03 | 79.2 |
| DBP | 8,462 | -0.05  (-0.13, 0.03) | 0.24 | 93.2 |
| Cholesterol | 8,438 | -0.002  (-0.05, 0.04) | 0.92 | 77.3 |
| HDL cholesterol | 8,213 | 0.07  (0.004, 0.13) | 0.04 | 87.9 |
| LDL cholesterol | 8,177 | 0.01  (-0.04, 0.06) | 0.73 | 78.0 |
| Triglycerides | 7,644 | -0.09  (-0.17, -0.01) | 0.04 | 92.0 |
| Glucose | 8,402 | -0.04  (-0.09, 0.01) | 0.16 | 83.0 |
| BMI | 8,448 | -0.05  (-0.13, 0.02) | 0.13 | 90.8 |
| WHR | 8,433 | -0.14  (-0.21, -0.07) | 0.0001 | 90.5 |
| Hypertension^2^ | 7,081 | 0.94  (0.83, 1.07) | 0.38 | 67.8 |

*Data are means (95% CI) for effect sizes per SD increase in L-ascorbic acid and meta-analysis P-values. All continuous traits are inverse-rank transformed before calculation of Z-score. All effect sizes (95% CIs) are presented in SD units. I^2^ is the percentage of total variance in study estimates that is due to between-study heterogeneity [*[*15*](#_ENREF_15)*].*

^1^*P-values are from random-effects meta-analysis of linear regression coefficients estimated within each study for each phenotype Z-score (on the inverse rank scale) against L-ascorbic acid Z-score (inverse rank scale).*

*^2^ Estimates for hypertension were calculated on the log(OR) scale, then exponentiated to give an expected OR estimate for hypertension risk.*

**Supplemental Table S6:** Meta-analysis of associations of cardiometabolic outcomes with *SLC23A1* variant rs33972313 and with L-ascorbic acid (excluding those with lipid-lowering and antihypertensive medication and the EPIC study)

| **Phenotype** | ***N*** | **Expected change in outcome Z-score per 0.23 SD L-ascorbic acid increase** | **Observed change in outcome Z-score per minor allele** | ***P*-value**  **(rs33972313 vs. outcome)^1^** | ***I^2^ (%)*** | ***P*-value for difference between observed and expected** |
| --- | --- | --- | --- | --- | --- | --- |
| SBP | 8,539 | -0.01  (-0.02, -0.0001) | -0.002  (-0.08, 0.08) | 0.96 | 0 | 0.85 |
| DBP | 8,539 | -0.01  (-0.03, 0.01) | -0.003  (-0.08, 0.08) | 0.95 | 0 | 0.87 |
| Cholesterol | 8,504 | -0.0004  (-0.01, 0.01) | -0.03  (-0.11, 0.06) | 0.54 | 0 | 0.55 |
| HDL cholesterol | 8,262 | 0.01  (0.001, 0.03) | -0.05  (-0.20, 0.09) | 0.45 | 66.2 | 0.35 |
| LDL cholesterol | 8,219 | 0.002  (-0.01, 0.01) | -0.03  (-0.11, 0.05) | 0.48 | 0 | 0.46 |
| Triglycerides | 7,710 | -0.02  (-0.03, -0.0005) | 0.09  (-0.07, 0.25) | 0.27 | 71.5 | 0.19 |
| Glucose | 8,470 | -0.01  (-0.02, 0.003) | 0.03  (-0.05, 0.11) | 0.45 | 0 | 0.33 |
| BMI | 8,523 | -0.01  (-0.02, 0.003) | 0.005  (-0.08, 0.09) | 0.91 | 13.4 | 0.75 |
| WHR | 8,504 | -0.03  (-0.04, -0.01) | -0.02  (-0.12, 0.09) | 0.75 | 37.7 | 0.87 |
| Hypertension^2^ | 7,025 | 0.98  (0.97, 1.01) | 1.05  (0.81, 1.35) | 0.73 | 3.8 | 0.67 |

*Data are means (95% CI) for observed and expected effect sizes and meta-analysis P-values. All continuous traits are inverse-rank transformed before calculation of Z-score. All effect sizes (95% CIs) are presented in SD units. I^2^ is the percentage of total variance in study estimates that is due to between-study heterogeneity [*[*15*](#_ENREF_15)*].*

^1^*P-values are from random-effects meta-analysis of linear regression within each study for each phenotype Z-score (on the inverse rank scale) against L-ascorbic acid Z-score (inverse rank scale).*

*^2^ Estimates for hypertension were calculated on the log(OR) scale, then exponentiated to give an expected OR estimate for hypertension risk.*

**Supplemental Table S7:** Meta-analysis of associations of cardiometabolic outcomes with *SLC23A1* variant rs33972313 and with L-ascorbic acid including the EPIC study

| **Phenotype** | ***N*** | **Expected change in outcome Z-score per effect allele** | **Observed change in outcome Z-score per effect allele** | ***P*-value**  **(rs33972313 vs. outcome)^1^** | ***I^2^ (%)*** | ***P*-value for difference between observed and expected** |
| --- | --- | --- | --- | --- | --- | --- |
| SBP | 16,789 | -0.02  (-0.04, -0.001) | 0.004  (-0.05, 0.06) | 0.88 | 0 | 0.50 |
| DBP | 16,789 | -0.02  (-0.04, 0.003) | -0.004  (-0.06, 0.05) | 0.90 | 0 | 0.69 |
| Cholesterol | 16,565 | -0.001  (-0.01, 0.01) | -0.02  (-0.07, 0.04) | 0.60 | 0 | 0.64 |
| HDL cholesterol | 16,112 | 0.02  (-0.01, 0.05) | -0.04  (-0.12, 0.05) | 0.39 | 44.3 | 0.15 |
| LDL cholesterol | 16,001 | -0.001  (-0.01, 0.01) | -0.02  (-0.08, 0.04) | 0.47 | 0 | 0.50 |
| Triglycerides | 15,460 | -0.03  (-0.05, -0.001) | 0.06  (-0.05, 0.16) | 0.28 | 62.2 | 0.10 |
| Glucose | 15,261 | -0.01  (-0.03, 0.003) | 0.02  (-0.04, 0.08) | 0.49 | 0 | 0.26 |
| BMI | 16,773 | -0.02  (-0.04, 0.002) | -0.005  (-0.06, 0.05) | 0.87 | 0 | 0.68 |
| WHR | 16,745 | -0.04  (-0.08, -0.003) | -0.03  (-0.08, 0.03) | 0.35 | 0 | 0.70 |
| Hypertension^2^ | 13,774 | 0.97  (0.93, 1.01) | 0.99  (0.84, 1.18) | 0.95 | 0 | 0.79 |

*Data are means (95% CI) for observed and expected effect sizes and meta-analysis P-values. All continuous traits are inverse-rank transformed before calculation of Z-score. All effect sizes (95% CIs) are presented in SD units. I^2^ is the percentage of total variance in study estimates that is due to between-study heterogeneity [*[*15*](#_ENREF_15)*].*

^1^*P-values are from random-effects meta-analysis of linear regression within each study for each phenotype Z-score (on the inverse rank scale) against L-ascorbic acid Z-score (inverse rank scale).*

*^2^ Estimates for hypertension were calculated on the log(OR) scale, then exponentiated to give an expected OR estimate for hypertension risk.*

**Supplemental Figures**

**Supplemental Figure S1:** Meta-analysis of the per-allele-effect of rs33972313 on L-ascorbic acid including the EPIC study

**Supplemental Figure S2:** Meta-analysis of per-allele-effect of rs33972313 on L-ascorbic acid

**Supplemental Figure S3:** Meta-analysis plot for key quantitative traits associated with L-ascorbic acid. Effect sizes (ES) are SD change in trait per 1-SD increase in L-ascorbic acid

**Supplemental Figure S4:** Associations between L-ascorbic acid and (A) SBP, (B) DBP, (C) Cholesterol, (D) HDL cholesterol, (E) LDL cholesterol, (F) Triglycerides, (G) Glucose, (H) BMI and (I) WHR

**A**

**B**

**C**

**D**

**E**

**F**

**G**

**H**

**I**

**Supplemental Figure S5:** Meta-analysis plot of key quantitative traits associated with L-ascorbic acid. Effect sizes (ES) are SD change in trait per 1-SD increase in L-ascorbic acid (including the EPIC study)

**Supplemental Figure S6:** Meta-analysis plot for key quantitative traits associated with *SLC23A1*. Effect sizes (ES) are SD change in trait per L-ascorbic acid increasing *SLC23A1* allele

**Supplemental Figure S7:** Associations between rs33972313 and (A) SBP, (B) DBP, (C) Cholesterol, (D) HDL cholesterol, (E) LDL cholesterol, (F) Triglycerides, (G) Glucose, (H) BMI and (I) WHR

**A**

**B**

**C**

**D**

**E**

**F**

**G**

**H**

**I**

**Supplemental Figure S8:** Meta-analysis plot of key quantitative traits associated with *SLC23A1*. Effect sizes (ES) are SD change in trait per L-ascorbic acid increasing *SLC23A1* allele (including the EPIC study)

**Supplemental Figure S9:** Observed effect estimates per *SLC23A1* allele for each cardiometabolic trait including the EPIC study, plotted against expected effect estimates, given the *SLC23A1*-L-ascorbic acid effect estimate and the observed L-ascorbic acid-trait associations. Error bars represent 95% CIs

**SBP**

**DBP**

**Cholesterol**

**HDL cholesterol**

**LDL cholesterol**

**Triglycerides**

**Glucose**

**BMI**

**WHR**

References

1. Timpson NJ, Forouhi NG, Brion MJ, Harbord RM, Cook DG, Johnson P, McConnachie A, Morris RW, Rodriguez S, Luan J, *et al.* Genetic variation at the SLC23A1 locus is associated with circulating concentrations of L-ascorbic acid (vitamin C): evidence from 5 independent studies with >15,000 participants. Am J Clin Nutr 2010; 92: 375-382.

2. Sargeant LA, Wareham N, Bingham S, Day NE, Luben RN, Oakes S, Welch A, Khaw KT. Vitamin C and hyperglycemia in the European Prospective Investigation into Cancer--Norfolk (EPIC-Norfolk) study: a population-based study. Diabetes Care 2000; 23: 726-732.

3. Livak KJ. Allelic discrimination using fluorogenic probes and the 5' nuclease assay. Genet Anal 1999; 14: 143-149.

4. Holland PM, Abramson RD, Watson R, Gelfand DH. Detection of specific polymerase chain reaction product by utilizing the 5'----3' exonuclease activity of Thermus aquaticus DNA polymerase. Proc Natl Acad Sci U S A 1991; 88: 7276-7280.

5. Hart CL, Deary IJ, Davey Smith G, Upton MN, Whalley LJ, Starr JM, Hole DJ, Wilson V, Watt GCM. Childhood IQ of parents related to characteristics of their offspring: linking the Scottish Mental Survey 1932 to the MIDSPAN Family Study. J Bios Sci 2005; 37: 623-639.

6. Talwar D, McConnachie A, Welsh P, Upton M, O'Reilly D, Davey Smith G, Watt G, Sattar N. Which circulating antioxidant vitamins are confounded by socioeconomic deprivation? The MIDSPAN family study. PLoS One 2010; 5: e11312.

7. Whincup PH, Owen CG, Sattar N, Cook DG. School dinners and markers of cardiovascular health and type 2 diabetes in 13-16 year olds: cross sectional study. BMJ 2005; 331: 1060-1061.

8. Whincup PH, Gilg JA, Owen CG, Odoki K, Alberti KG, Cook DG. British South Asians aged 13-16 years have higher fasting glucose and insulin levels than Europeans. Diabet Med 2005; 22: 1275-1277.

9. Whincup PH, Cook DG, Adshead F, Taylor S, Papacosta O, Walker M, Wilson V. Cardiovascular risk factors in Britist children from towns with widely differing adult cardiovascular mortality. BMJ 1996; 313: 79-84.

10. Emberson JR, Whincup PH, Morris RW, Walker M. Re-assessing the contribution of serum total cholesterol, blood pressure and cigarette smoking to the aetiology of coronary heart disease: impact of regression dilution bias. Eur Heart J 2003; 24: 1719-1726.

11. Thelle DS, Shaper AG, Whitehead TP, Bullock DG, Ashby D, Patel I. Blood lipids in middle-aged British men*.* Br Heart J 1983; 49: 205-213.

12. Shaper AG, Pocock SJ, Walker M, Cohen NM, Wale CJ, Thomson AG. British Regional Heart Study: cardiovascular risk factors in middle-aged men in 24 towns. BMJ 1981; 283: 179-186.

13. Emberson JR, Whincup PH, Walker M, Thomas M, Alberti KG. Biochemical measures in a population-based study: effect of fasting duration and time of day. Ann Clin Biochem 2002; 39: 493-501.

14. Wannamethee SG, Papacosta O, Lawlor DA, Whincup PH, Lowe GD, Ebrahim S, Sattar N. Do women exhibit greater differences in established and novel risk factors between diabetes and non-diabetes than men? The British Regional Heart Study and British Women's Heart Health Study. Diabetologia 2012; 55: 80-87.

15. Higgins JP, Thompson SG, Deeks JJ, Altman DG. Measuring inconsistency in meta-analyses. BMJ 2003; 327: 557-560.
